# How do we walk and chew gum at the same time?

**DOI:** 10.7554/eLife.03235

**Published:** 2014-06-03

**Authors:** Philippe Morquette, Arlette Kolta

**Affiliations:** 1 **Philippe Morquette** is in the Groupe de Recherche sur le système Nerveux Central and the Département de Neurosciences, Université de Montréal, Montréal, Canadaphilippe.morquette@umontreal.ca; 2 **Arlette Kolta** is in the Groupe de Recherche sur le système Nerveux Central, the Faculté de Médecine Dentaire and the Département de Neurosciences, Université de Montréal, Montréal, Canadaarlette.kolta@umontreal.ca

**Keywords:** premotor circuit, monosynaptic rabies virus, jaw motoneurons, tongue motoneurons, orofacial coordination, Mouse

## Abstract

A genetic approach has been used to map the neural circuits that control and coordinate the tongue and jaw muscles.

**Related research article** Stanek E IV, Cheng S, Takatoh J, Han B-X, Wang F. 2014. Monosynaptic premotor circuit tracing reveals neural substrates for oro-motor coordination. *eLife* **3**:e02511. doi: 10.7554/eLife.02511

**Image** Premotor neurons can project to motoneurons that control the tongue, the jaw muscles or both.

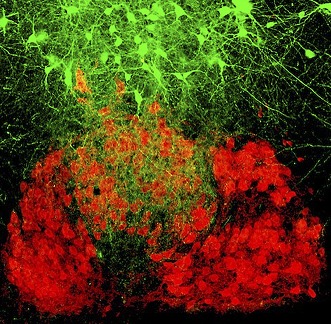

President Lyndon Johnson is reported to have said that Gerald Ford—then a congressman, later the President—was ‘so dumb that he can't walk and chew gum at the same time’. What Johnson actually said was somewhat cruder, but his message was the same: the inability to perform two tasks simultaneously is a sign of limited intelligence. Motor coordination does not actually depend on intelligence, but it does not happen automatically. It might seem trivial to coordinate two simple tasks such as walking and chewing, but as we grow older, talking while walking becomes increasingly difficult (Lundin-Olsson et al., 1997). And it is even more difficult to simultaneously execute two movements using the same group of muscles, such as talking and chewing for instance.

For decades, neuroscientists have tried to understand how neural circuits are configured so as to allow flexibility and versatility, while also ensuring proper coordination and avoiding conflicts (such as accidentally biting one's tongue while talking or eating). This question was first tackled in the neural circuits responsible for simple rhythmic behaviours (such as breathing, walking or chewing) that are executed automatically. The circuits controlling these movements are called central pattern generators (Marder and Calabrese, 1996), and each rhythmic behaviour—and perhaps every muscle—has its own central pattern generator. However, it is not clear how these generators interact in order to coordinate with each other when several different muscles are involved in a specific behaviour, or when a particular muscle is involved in several different behaviours.

Previous work in invertebrate systems suggests that at least two types of neural architectures exist: dedicated circuits and multifunctional circuits. Dedicated circuits generate a specific behaviour and, once activated, they shut off all other circuits that could generate other behaviours using the same muscles. Multifunctional circuits, on the other hand, contain various building blocks and are involved in more than one type of behaviour, although not all building blocks are involved in all behaviours (Morton and Chiel, 1994; Briggman and Kristan, 2008).

The operations performed by a neuronal circuit depend on the properties of its constituents (the neurons) and on their pattern of interconnectivity. Motoneurons are the neurons that transmit motor commands from the central nervous system to the muscles. Thus, to understand how a motor circuit operates, one must first identify the premotor neurons that are connected to the motoneurons. However, even the circuits controlling simple rhythmic behaviours in invertebrates turned out to be remarkably complex (Selverston and Moulins, 1985), and investigations of the circuits responsible for coordinating more complex behaviours in mammals have been hampered by technical limitations until recently.

Now, in *eLife*, Fan Wang and colleagues at Duke University—including Edward Stanek as first author—report the use of an elegant genetic approach to map the connectivity of the circuits controlling the tongue and jaw muscles in an effort to understand how orofacial movements are coordinated during behaviours such as chewing, swallowing and talking (Stanek et al., 2014). They wanted to learn more about the sequence of commands for activating the relevant motoneurons while avoiding problems such as biting your tongue or choking when swallowing.

Stanek et al. used genetic engineering to generate mice that expressed a rabies glycoprotein in all of their motoneurons. They then injected various facial muscles in the mice with a modified rabies virus that contained a fluorescent tag instead of this glycoprotein. The virus entered the motoneurons that drive the muscle and, when complemented by the glycoprotein there, the virus was able to cross the synapse between the motoneurons and the premotor neurons connected to them (Wickersham et al., 2007). By sectioning the brainstem and illuminating these sections with a laser to visualize the fluorescence, it was possible to identify the premotor neurons that projected to various groups of motoneurons. Stanek et al. actually used two types of modified virus, one labelled with a red fluorescent tag and one labelled with a green fluorescent tag.

Many of their findings confirmed results obtained with conventional tracing methods, but they also showed that some premotor neurons projected to more than one set of motoneurons. For instance, in one set of experiments they injected the red viruses into the muscle that closes the jaw (the masseter muscle) on one side of the face, and the green viruses into the masseter muscle on the other side of the face (Figure 1A). Three different sets of premotor neurons could be seen when the fluorescence was imaged: some premotor neurons projected to the motoneurons that drive the masseter muscle on the left side of the face, others projected to the motoneurons that drive the other masseter muscle, and some premotor neurons projected to both sets of motoneurons. These premotor neurons are responsible for synchronizing the activation of the motoneurons that drive the masseter muscles on different sides of the face

**Figure 1.**
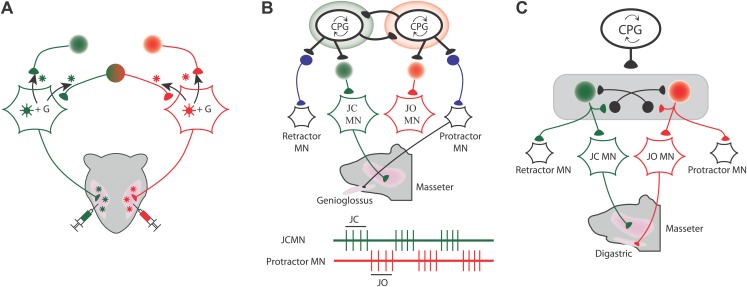
Mapping the neural circuits responsible for orofacial coordination. (**A**) When viruses labelled with green and red fluorescent tags are injected into the left and right muscles that close the jaw, three different sets of premotor neurons can be seen when the fluorescence is imaged: those that project to the motoneurons that drive the muscle on the left (green); those that project to the motoneurons that drive the muscle on the right (red); and those that project to both sets of motoneurons in order to coordinate the movements of the two muscles (green and red). The virus can move across the synapse between the motoneurons and the premotor neurons when it is complemented by a glycoprotein (**G**) that is expressed in the motoneurons of the mice: this movement is indicated by the black arrows. (**B** and **C**) During chewing, the digastric muscles (which open the jaw) and the genioglossus muscle (which protracts the tongue) are active during the jaw opening (JO) phase, while the masseter muscles (which close the jaw) and the muscle that retracts the tongue are both active during the jaw closing (JC) phase. The green and red traces show that the jaw closing motoneurons (JCMN) and the protractor motoneurons (protractor MN) are active at different times. The results of Stanek et al. suggest that orofacial coordination is performed by multifunctional circuits rather than dedicated circuits. In dedicated circuits (**B**), motoneurons do not share their premotor neurons, and those that are active at the same time receive inputs from their central pattern generator (CPG), which inhibits activity of other circuits. In multifunctional circuits (**C**), sets of motoneurons driving different muscles can share their premotor neurons. The same CPG activates premotor neurons that are shared by the motoneurons that are active at the same time, and this same CPG also inhibits (as indicated by the black neurons that end in a black circle) the premotor neurons that project to motoneurons that are active at other times.

FIGURE CREDIT: PHILIPPE MORQUETTE

Stanek et al. found similar patterns when they injected viruses into other pairs of muscles. For example, some premotor neurons projected to motoneurons that drive the masseter muscle and also to motoneurons that drive the muscle that retracts the tongue. Similarly, some premotor neurons projected to motoneurons that drive the digastric muscle (which opens the jaw) and also to motoneurons that drive the muscle that protracts the tongue. However, the muscle that protracts the tongue did not share any premotor neurons with the muscle that closes the jaw. Likewise, the muscle that retracts the tongue did not share any premotor neurons with the muscle that opens the jaw. Significantly, however, all these premotor neurons were found in the same areas of the brain, which indicates that they probably receive commands from the same central pattern generator and not from two different central pattern generators. These results argue against purely dedicated circuitry and are more suggestive of a multifunctional scheme (Figure 1B,C).

While this study illuminated the anatomical framework for orofacial circuitry, the connectivity of a network only defines the constraints within which it operates; it does not tell us how the network functions. That said, this new technology will open the door to a myriad of studies linking structure to function. Moreover, in addition to fluorescent tags, modified viruses could be used to carry other molecules to specific premotor neurons. These molecules could be used to tell us more about the target neurons: for example, they could tell us when and how the neurons are active. Furthermore, they could be used to modify the activity of the target neurons or to influence gene expression in these neurons (Osakada et al., 2011).
